# Staged Emergency Caesarean Section and Cementless Total Hip Arthroplasty Within a Single Anaesthetic Episode for Pipkin Type III Femoral Head Fracture-Dislocation in a Gravid Patient: A Case Report

**DOI:** 10.7759/cureus.111429

**Published:** 2026-06-24

**Authors:** Tamer Farouk Elsaid, Mohamed E Eldesouky, Hanan A Bahaaeldin, Zeinab Al-Rawi, Francisco Chana Rodriguez

**Affiliations:** 1 Trauma and Orthopaedics, Rashid Hospital, Dubai, ARE; 2 Orthopaedics and Traumatology, Mohammed Bin Rashid University of Medicine and Health Sciences, Dubai, ARE; 3 Diagnostic Radiology, Zagazig University, Zagazig, EGY; 4 Diagnostic Radiology, Emirates Hospital, Dubai, ARE

**Keywords:** cementless total hip arthroplasty, multidisciplinary management, pipkin type iii fracture, pregnancy, sciatic nerve compression, trauma in pregnancy

## Abstract

Pipkin type III femoral head fracture-dislocation in a gravid patient is an exceptionally rare clinical entity. We report the case of a woman in her early 40s who presented at 36 weeks of gestation following a high-energy motor vehicle collision. Clinical examination and imaging confirmed a left posterior hip dislocation; computed tomography revealed a severely comminuted Pipkin type III fracture that precluded open reduction and internal fixation. A multidisciplinary team formulated a staged single-anaesthetic operative strategy; emergency caesarean section was performed immediately before cementless total hip arthroplasty (THA) within the same anaesthetic episode. A live male neonate was delivered (Apgar scores 3, 6, and 7 at one, five, and 10 minutes). Intraoperatively, the dislocated femoral head was confirmed in direct apposition with the sciatic nerve. THA was completed successfully via a posterolateral approach. Total estimated blood loss was 1,900 mL, requiring transfusion of six units of packed red blood cells. The patient achieved full weight-bearing on postoperative day three and was discharged neurologically intact after eight days. At the three-month follow-up, the Merle d'Aubigné score was 17/18. This case, supported by a focused literature review, demonstrates that a staged same-anaesthetic strategy can achieve favourable maternal and neonatal outcomes in this rare, high-stakes clinical scenario and provides a practical decision-making framework for multidisciplinary teams.

## Introduction

Traumatic hip dislocation accounts for approximately 2-5% of all joint dislocations and is most commonly caused by high-energy mechanisms such as motor vehicle collisions [1]. Its occurrence in a gravid patient is considerably rare, with only isolated cases reported in the peer-reviewed literature [2]. The Pipkin classification describes four subtypes of femoral head fracture associated with hip dislocation [1,3,4]: type I (fracture inferior to the fovea capitis), type II (fracture at or superior to the fovea), type III (femoral head fracture with concurrent femoral neck fracture), and type IV (any of the above with an associated acetabular fracture).

Pipkin type III injuries are the least common subtype and carry the worst prognosis for femoral head preservation [4,5]. The concurrent neck fracture disrupts the medial circumflex femoral artery, which is the principal blood supply to the femoral head, rendering avascular necrosis (AVN) near-inevitable if the head is retained. When extensive comminution precludes open reduction and internal fixation (ORIF), primary total hip arthroplasty (THA) represents the definitive intervention [6-9].

Pregnancy introduces a constellation of competing priorities: anaesthetic risk, radiation exposure, foetal haemodynamic stability, uterine contractility, and sequelae of high-energy trauma, including placental abruption and preterm labour, must be simultaneously managed [10-12]. Elective THA is generally deferred until postpartum; however, posttraumatic cases with non-reconstructable fractures may necessitate urgent arthroplasty [1,5]. A combined caesarean section and THA strategy within a single anaesthetic episode, whilst rare, may be appropriate when the patient is near term and arthroplasty is urgently required [8]. Neurovascular compromise from sciatic nerve impingement by a dislocated femoral head further escalates operative urgency [10].

We report a case of a gravid patient at 36 weeks of gestation who sustained a Pipkin type III posterior hip fracture-dislocation following a motor vehicle collision, successfully treated via a staged approach comprising emergency caesarean section immediately followed by cementless THA. The management principles and decision-making framework applied in this case are further discussed in the context of a focused literature review on THA in pregnancy.

## Case presentation

A woman in her early 40s, gravida 4 para 3, with three prior uncomplicated vaginal deliveries at presentation and no significant past orthopaedic history, presented at 36 weeks of gestation after a rear-end and left-sided motor vehicle collision. She reported severe left hip pain (8/10 on the Numerical Rating Scale) [13], with visible lower-limb deformity. Primary and secondary surveys followed Advanced Trauma Life Support (ATLS) principles. The cardiovascular and respiratory systems were haemodynamically stable. The gravid abdomen showed no external trauma. Cardiotocography demonstrated regular foetal heart tones, followed by mild uterine contractions.

Examination of the left lower limb revealed shortening, internal rotation, and adduction, the classical triad of posterior hip dislocation. Active and passive range of motion was markedly restricted (flexion 60°, adduction 20°). Distal pulses were intact bilaterally. Neurological assessment demonstrated preserved motor function; however, paraesthesia over the left posterior thigh and leg raised concern for sciatic nerve involvement. No open wounds were identified. 

Anteroposterior (AP) radiography of the pelvis was obtained in the Emergency Department and confirmed left posterior hip dislocation with superior lateral femoral head displacement and disruption of Shenton's line (Figure 1).

**Figure 1:**
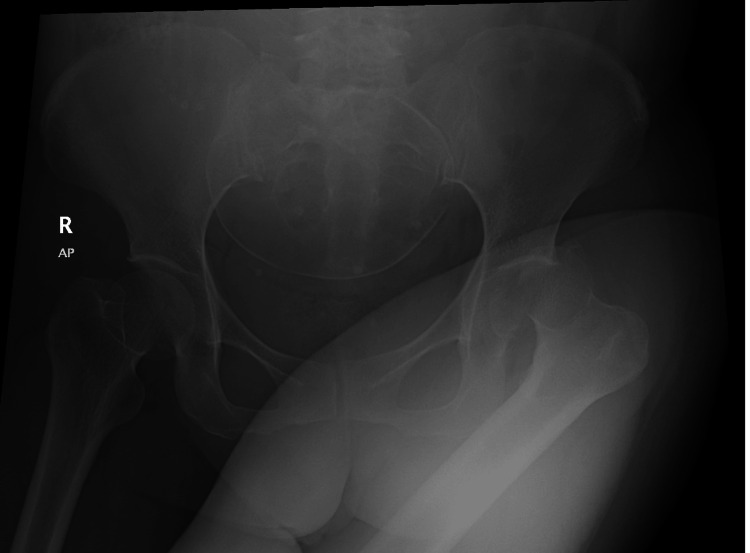
Pelvis radiograph (anteroposterior view) showing left posterior hip dislocation.

An initial attempt at closed reduction under conscious sedation was unsuccessful. Multi-planar CT of the pelvis, including axial, coronal, and sagittal reconstructions, was subsequently performed. CT demonstrated severe comminution of the femoral head with multiple fracture fragments. Crucially, a fracture extending through the femoral neck was identified, fulfilling the criteria for Pipkin type III classification (Figure 2) [6,7].

**Figure 2:**
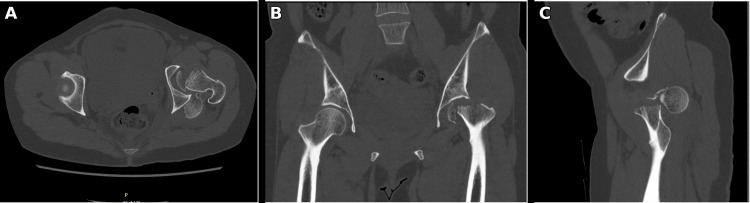
CT pelvis multiplanar reconstructions (Bone Window). (A) Axial: posteriorly dislocated, comminuted femoral head. (B) Coronal: femoral neck fracture confirming Pipkin type III classification. (C) Sagittal: proximity of the dislocated femoral head to the sciatic nerve.

A multidisciplinary team comprising anaesthesia, obstetrics, neonatology, and orthopaedic trauma teams convened urgently. The following factors drove the decision for immediate combined surgery (ordered by priority): (i) failed closed reduction; (ii) Pipkin type III fracture with non-reconstructable comminution confirmed on CT; (iii) advancing uterine contractions at 36 weeks' gestation; (iv) progressive sciatic nerve paraesthesia from femoral head compression; and (v) risk of placental compromise with further delay.

A staged single-anaesthetic operative strategy was agreed. This included (i) Stage 1: Emergency Caesarean Section: Performed in supine position under general anaesthesia to eliminate foetal exposure to the physiological stress of a prolonged orthopaedic procedure, to arrest uterine contractions, and to decompress the inferior vena cava, thereby optimising maternal cardiac output for the subsequent THA; (ii) Stage 2: Urgent Cementless THA: Performed immediately following caesarean section within the same anaesthetic episode to urgently decompress the sciatic nerve as a priority avoiding permanent sciatic nerve damage in addition to avoiding a second general anaesthetic in a haemodynamically compromised postpartum patient.

The caesarean section was completed without intraoperative complications. A live male neonate was delivered with Apgar scores of 3, 6, and 7 at one, five, and 10 minutes, respectively [14]. The neonate was transferred to the neonatal intensive care unit (NICU) for routine monitoring in the context of preterm gestation (36 weeks) and a depressed initial Apgar score consistent with the effects of maternal general anaesthesia; resuscitation details and NICU course were managed exclusively by the neonatology team and were not available for review at the time of this report, representing a recognised limitation of this case documentation. Neonatal status at the time of maternal discharge was documented as satisfactory in the patient's medical record.

Without delay, THA was commenced with the patient turned to the lateral decubitus position, with sterilization of the field and using a posterolateral approach. Intraoperative findings confirmed severe femoral head comminution and the dislocated femoral head in direct apposition with the sciatic nerve (Figure 3), consistent with the pre-operative neurological findings. All comminuted femoral head fragments were meticulously excised. A cementless Trident II Tritanium acetabular cup size 50 (Stryker Corporation, Portage, Michigan, United States) was inserted with 10 degrees anteversion with optimal press-fit stability and supplementary 6.5 mm screw fixation. An Accolade II cementless femoral stem size 4 (Stryker Corporation) was impacted with 10 degrees of anteversion and confirmed stable at primary fixation. A universal ceramic head on a polyethylene bearing surface was used. Wounds were irrigated and closed in layers.

**Figure 3:**
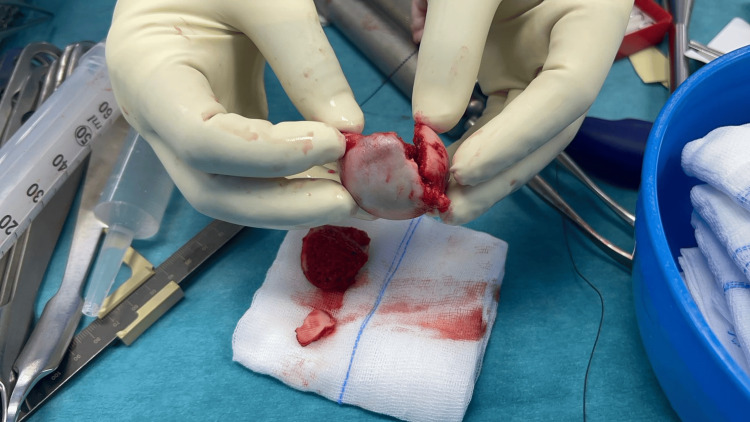
Intraoperative photograph. Comminuted femoral head fragments.

Total estimated blood loss from both combined procedures was 1,900 mL (1,200 ml from caesarean section and 700 ml from THA), requiring transfusion of six units of packed red blood cells in accordance with the institutional massive transfusion protocol; Tranexamic acid was not administered; the decision was based on the absence of clinical or haematological evidence of hyperfibrinolysis and concerns regarding potential effects on uteroplacental perfusion in the immediate peripartum period.

Postoperatively, the patient was admitted to the intensive care unit. She developed sinus tachycardia and a hypertensive crisis (blood pressure 232/154 mmHg). Postpartum pre-eclampsia was suspected; Laboratory investigations performed postoperatively are shown in Table 1.

**Table 1:** Postoperative laboratory investigations for pre-eclampsia evaluation. NT-proBNP: N-terminal pro-brain natriuretic peptide; FEU: fibrinogen equivalent units

Parameter	Patient Value	Reference Range	Interpretation
Urinary albumin/creatinine ratio	16.5 mg/g	< 30 mg/g	Normal — no proteinuria
NT-proBNP	76.4 pg/mL	< 125 pg/mL (age < 75 yrs)	Normal — no cardiac strain
D-dimer	18.6 μg/mL FEU	< 0.5 μg/mL (non-pregnant); up to 3.0 μg/mL (third trimester)	Elevated — attributed to recent major surgery and delivery
CT Pulmonary Angiography	Negative	No filling defect	Pulmonary embolism excluded

In the absence of proteinuria or biochemical evidence of end-organ dysfunction, a formal diagnosis of pre-eclampsia could not be established, and the hypertensive crisis was attributed to postpartum hypertension in the context of physiological stress, pain, and blood loss from combined major surgery. Two days of intensive care monitoring were followed by three days in a high-dependency unit and three days in a standard orthopaedic ward. Physiotherapy commenced on postoperative day one with passive range-of-motion exercises. 

Preoperatively, intravenous cefazolin 2 g was administered. Thromboprophylaxis was initiated with enoxaparin 40 mg subcutaneously once daily and continued for 35 days per institutional protocol for combined major obstetric and orthopaedic surgery.

Full weight-bearing with a walking frame was achieved on postoperative day three. Postoperative radiographs confirmed optimal positioning of both acetabular and femoral components, with satisfactory offset and leg-length symmetry (Figure 4). Neurovascular examination at discharge (postoperative day 8) documented intact motor and sensory function with complete resolution of pre-operative paraesthesia.

**Figure 4:**
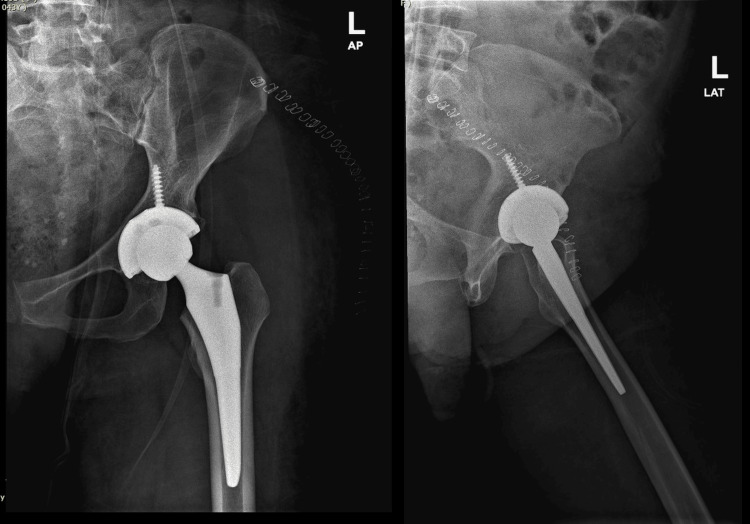
Postoperative left hip radiograph showing optimal cementless THA positioning. (A) Anteroposterior view; (B) Lateral view THA: total hip arthroplasty

The patient was discharged with a structured physiotherapy program and outpatient follow-up at six weeks, three months, and one year. At the three-month follow-up, the Merle d’Aubigné score was 17/18 (pain 6, mobility 5, gait 6) [15], indicating an excellent functional outcome in the context of short follow-up data. No subsidence of implants was observed (Figure 5).

**Figure 5:**
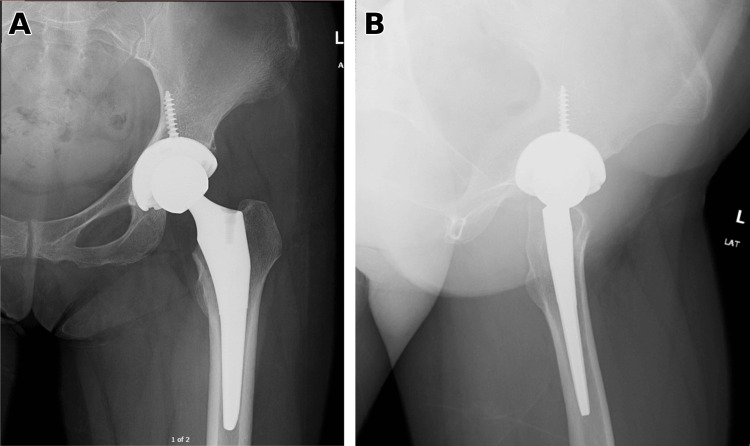
Left hip radiographs at the three-month follow-up showing proper implant position. (A) Anteroposterior view; (B) Lateral view

## Discussion

This case illustrates an exceptionally rare clinical scenario: Pipkin type III femoral head fracture-dislocation in a patient at 36 weeks of gestation. To the best of our knowledge, this represents one of the few published cases in which primary cementless THA was performed as part of a staged, single anaesthetic episode strategy with emergency caesarean section. We discuss the key decision points and integrate a focused literature review to rationalize our management.

Rationale for primary THA

Pipkin type III injuries carry the worst prognosis for femoral head preservation [1,4,5]. The concurrent femoral neck fracture disrupts the medial circumflex femoral artery, rendering AVN near-inevitable if the head is retained. Extensive intraoperative comminution confirmed that ORIF was not feasible, providing unequivocal justification for immediate THA consistent with published evidence [6-9]. The literature consistently supports that in posttraumatic cases where fixation is not achievable, primary THA is the appropriate definitive intervention.

THA in pregnancy: literature review

Table 2 summarises the evidence-based principles of THA management in pregnancy derived from a focused literature review, which directly informed our multidisciplinary decision-making.

**Table 2:** Evidence-based principles of THA in pregnancy. THA: total hip arthroplasty; AVN: avascular necrosis; CTG: cardiotocography; NSAIDs: non-steroidal anti-inflammatory drugs; IV: intravenous

Category	Principle	Key Considerations	References
General indication	Defer elective THA until postpartum unless trauma is severe and limb-threatening	Conservative management: bed rest, pelvic binder, skin traction, temporary external fixation	[5,1]
Posttraumatic indication	Urgent THA if conservative management fails or the fracture is non-reconstructable	AVN, displaced neck fracture, or severe joint destruction may mandate urgent arthroplasty	[16,5]
First trimester	Avoid surgery unless it's life-threatening	The highest risk of teratogenicity and miscarriage	[5,8]
Second trimester	Safest window for non-obstetric surgery	Organogenesis is complete; reduced preterm labour risk	[5,8]
Third trimester	Postpone unless emergent	High preterm labour risk; continuous foetal monitoring required	[5,8,10]
Multidisciplinary team	Essential for optimal outcomes	Orthopaedic surgeon, obstetrician, anaesthesiologist, neonatologist	[5,8]
Anaesthesia	Regional preferred when feasible	Reduces foetal exposure to general anaesthetic agents	[5,10]
Foetal monitoring	Continuous intraoperative CTG at viable gestation	Cardiotocography during and after procedure	[10]
Combined C-section + THA	Rare; consider if near term and THA urgently required	C-section first, then THA in same anaesthetic episode to avoid multiple exposures	[8]
Haemodynamic support	Essential for combined procedures	IV fluids, blood products, extended operative planning	[10]
Thromboprophylaxis	Mandatory — dual thrombotic risk	Enoxaparin or fondaparinux; avoid NSAIDs in late pregnancy	[5,1]
Pain management	Paracetamol-based multimodal analgesia	Avoid NSAIDs in third trimester (renal and amniotic fluid risk)	[1]
Mobilisation	Early ambulation postpartum	Full weight-bearing targeted by 6 weeks post-op	[8]

Comparison With Published Cases

Table 3 compares our case with the limited published literature on hip fractures in pregnancy requiring arthroplasty. Unlike previously reported cases where initial fixation failed before arthroplasty was ultimately required, our patient underwent definitive THA as the primary procedure, avoiding the morbidity of a failed fixation attempt and a further anaesthetic [6,16]. Compared with a case where THA was deferred three days postpartum [7], our approach eliminated three further days of sciatic nerve compression and hip instability, contributing to complete neurological recovery at discharge.

**Table 3:** Similar cases of hip fracture in pregnancy requiring arthroplasty. THR: total hip replacement; THA: total hip arthroplasty; AVN: avascular necrosis; C-section: caesarean section.

Age (years)	Gestation/Delivery	Fracture	Treatment	Case Report
24	34 weeks / C-section	Bilateral femoral neck fractures	R: dynamic hip screws; L: THR after failed fixation	Wright et al., 2021 [16]
38	35 weeks / C-section	Right femoral neck fracture	THR delayed 3 days postpartum	Klimko et al. 2021 [7]
Early 40s (present)	36 weeks / C-section	Pipkin type III femoral head fracture-dislocation (L)	Staged: emergency C-section then immediate cementless THA — single anaesthetic	Current report

Staged Operative Strategy

Delaying foetal delivery would have exposed the neonate to the physiological insults of a prolonged orthopaedic procedure, including haemodynamic shifts and significant blood loss. Conversely, deferring THA after caesarean section would have required a second general anaesthetic in a haemodynamically compromised postpartum patient, whilst prolonging sciatic nerve compression and hip instability [10]. The same-session staged strategy mitigated both risks effectively, consistent with published guidance that combined procedures may be considered when the patient is near term, and arthroplasty is urgently required [8].

Sciatic Nerve Compression

Sciatic nerve compression by a posteriorly dislocated femoral head is a recognised complication and an independent urgency indicator for surgical intervention [1,2]. In the present case, paraesthesia over the posterior thigh and leg was corroborated by direct intraoperative visualisation of nerve-head apposition via posterolateral approach. Decompression via femoral head excision resulted in complete neurological recovery at discharge, an outcome consistent with the literature supporting early decompression [1,2].

Radiation Safety

Pelvic CT exposes the foetus to approximately 25-79 mGy, well below the 100 mGy threshold for deterministic foetal effects [17]. Stochastic risks at these doses increase baseline childhood malignancy risk by less than 0.1% [10]. In this case, CT was indispensable for fracture characterisation, Pipkin classification, and operative planning, and its diagnostic value substantially outweighed the minimal theoretical foetal risk.

Haemodynamic and Obstetric Management

The combined estimated blood loss of 1,900 mL reflects the additive haemorrhagic risk of major orthopaedic surgery and caesarean section in a polytraumatised patient [10, 12]. Elective THA is typically associated with 700-1500 mL blood loss; the high-energy mechanism, hyperaemic gravid state, and haemostatic disturbances likely amplify haemorrhage. The postoperative hypertensive crisis (232/154 mmHg) underscores the need for continued obstetric vigilance in the postoperative period [10,11]. 

Cementless THA in a Young Patient

Cementless THA was selected, given the patient's relatively young age and high activity expectations. Contemporary literature consistently demonstrates superior long-term biological fixation, lower revision rates, and comparable or better functional outcomes with cementless constructs in younger cohorts [18,19]. Early full weight-bearing on postoperative day three is consistent with cementless press-fit principles and rapid-recovery THA protocols.

Role of Tranexamic Acid

Tranexamic acid was considered but not administered due to the absence of hyperfibrinolysis on rotational thromboelastometry and concerns regarding placental perfusion in the immediate pre-delivery period. Current evidence supports tranexamic acid use in postpartum haemorrhage and major trauma, but its safety in combined caesarean-arthroplasty cases remains unestablished [20].

Limitations of this case report

The key limitations to acknowledge in this report include: a single case study, a short functional follow-up for three months, and no long-term implant data for proper cementless THA osseointegration assessment. In addition, neonatal resuscitation details and NICU course data, which were managed by the neonatology team and were not available for retrospective review, represent a limitation inherent to single-specialty orthopaedic case documentation.

## Conclusions

The key lessons from this case are as follows. First, Pipkin type III injuries with extensive comminution mandate primary THA, as ORIF is not feasible and retention of the femoral head carries a near-certain risk of avascular necrosis. Second, in a gravid patient near term, a staged same anaesthetic approach emergency caesarean section immediately followed by THA avoids foetal exposure to prolonged orthopaedic surgery and eliminates the need for a second anaesthetic. Third, sciatic nerve compression by a posteriorly dislocated femoral head is an independent urgency indicator, and prompt surgical decompression achieved complete neurological recovery at short-term follow-up in this case. Fourth, pelvic CT is clinically justified in pregnancy when essential for operative planning, as foetal dose remains well below accepted deterministic thresholds. Fifth, postoperative obstetric vigilance, particularly for pre-eclampsia, must continue into the intensive care period, as multidisciplinary collaboration beyond the operating theatre is as critical as intraoperative coordination.
